# Sirt5-mediated desuccinylation of OPTN protects retinal ganglion cells from autophagic flux blockade in diabetic retinopathy

**DOI:** 10.1038/s41420-022-00861-5

**Published:** 2022-02-14

**Authors:** Ye Zhang, Tingting Li, Xuan Cai, Da Long, Xiangning Wang, Chang Liu, Qiang Wu

**Affiliations:** Department of Ophthalmology, Shanghai Jiao Tong University Affiliated Sixth People’s Hospital, Shanghai, 200233 China

**Keywords:** Diabetes complications, Retinal diseases, Macroautophagy, Post-translational modifications

## Abstract

Retinal neurodegeneration develops early in the course of diabetic retinopathy (DR), and our previous research showed that succinate accumulation results in retinal ganglion cells (RGCs) dysfunction in the retinas of rats with DR. Succinate can enhance lysine succinylation, but the succinylation of DR is not well understood. In this study, we investigated the role of the succinylome in DR and identified the key factor in this process. TMT labeling and LC–MS/MS analysis were combined to quantify the differentially succinylated proteins between vitreous humor (VH) samples from DR and non-DR patients. A total of 74 sites in 35 proteins were differentially succinylated between DR and non-DR vitreous humor samples, among which succinylation of the K108 site of optineurin (OPTN K108^su^) in the defense response was enriched by GO analysis based on the biological process category. Then, using a streptozotocin (STZ)-induced diabetic rat model, R28 cells and primary rat RGCs (rRGCs), we demonstrated that OPTN underwent lysine succinylation in the retinas of rats with DR and that OPTN K108^su^ mediated autophagic flux blockade under high-glucose (HG) conditions. Sirt5 can desuccinylate OPTN K108^su^, thus protecting RGCs function from high glucose-induced RGCs autophagic flux blockade in the diabetic retina. Overall, desuccinylation of OPTN is an essential adaptive mechanism for ameliorating autophagic flux blockade in RGCs under DR conditions, and targeting the Sirt5-desuccK108-OPTN axis may thus open an avenue for therapeutic intervention in RCGs dysfunction.

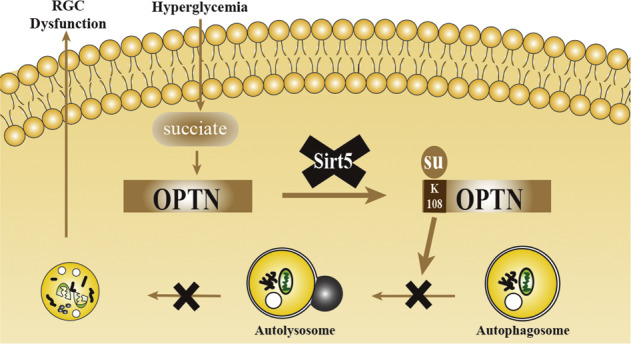

## Introduction

Diabetic retinopathy (DR) is a neural and microvascular complication of diabetes mellitus (DM) and remains the leading cause of preventable blindness in working-aged people [1]. Recent research has demonstrated that neurodegenerative processes develop early in the course of DR, even before the onset of microvascular changes [2–4]. Neurons, primarily retinal ganglion cells (RGCs) in the inner retinal layers, first lose adaptability to blood glucose alterations caused by diabetes, as evidenced by apoptotic cell death and retinal layer thinning in human and animal models [5]. Although the underlying mechanism of early RGCs dysfunction in DR remains to be clarified, understanding the early effects of diabetes on neural cells in the retina could eventually lead to the early identification and diagnosis of DR.

Our previous studies demonstrated that an accumulation of succinate in streptozotocin (STZ)-induced type 1 diabetes mellitus (T1DM) rat retinas promotes RGCs dysfunction in the early stage of DR [6]. Succinate, an important metabolite of the tricarboxylic acid cycle, can significantly enhance lysine succinylation [7]. Succinylation is a newly discovered posttranslational modification [7] in which a succinyl group (-CO-CH2-CH2-CO2H) is bound to a lysine residue through enzymatic or nonenzymatic means [8]. Aberrant succinylation is closely related to the occurrence and development of various diseases, including tumors, metabolic diseases and nervous system diseases [9], but its role in DR remains undefined. In this study, we analyzed the succinylome in the human vitreous humor (VH) samples to systematically identify differences between patients with and without DR. In our results, differentially succinylated proteins regulating various biological processes were identified, with particular enrichment of defense response; among these proteins, the succinylation of optineurin (OPTN) particularly interested us and was also a previously unreported modification of OPTN.

OPTN, the most recently identified autophagy receptor, was initially named because it corresponds to one of the genes encoding the glaucoma form of the “optic neuropathy inducing” protein [10]. OPTN has been implicated genetically in many human diseases, such as glaucoma, Paget’s disease and amyotrophic lateral sclerosis (ALS) [11]. In the retina, OPTN is preferentially expressed in RGCs [12]. The OPTN_M98K_ mutant [13] or OPTN_E50K_ mutant [14] induces autophagic death of RGCs in glaucoma, but its relationship with DR has not been determined conclusively. In addition, the posttranslational modification of OPTN is also closely related to the autophagy process; for example, TBK1-mediated phosphorylation of OPTN activates autophagy to clear invading Salmonella [15, 16], and HACE1-mediated ubiquitylation of OPTN promotes autophagic flux to suppress tumorigenicity in lung cancer cells [17]. Autophagy is a continuous and dynamic process in which cells produce autophagosomes under stress and deliver damaged proteins or organelles to the lysosome for degradation. This continuous process is called autophagic flux. OPTN contributes to both the formation of autophagosomes [15, 16] and the autophagosome-lysosome fusion [18] stage in autophagic flux. Many studies have reported that high glucose (HG)-induced autophagic flux blockade in retinal neurocytes, which is marked by the accumulation of autophagosomes and the substrate p62/SQTSM1, is an early event in the pathogenesis of DR neurodegeneration [19]. Given that OPTN plays a critical role in neurological and autophagic functions, we investigated the impact and mechanism of OPTN succinylation in RGCs autophagic flux blockade.

Protein succinylation is regulated by sirtuins (Sirt) [20], which belong to the histone deacetylase family and have been widely studied for their nicotinamide adenine dinucleotide (NAD^+^)-dependent deacetylase activity. Among members of the sirtuin family, Sirt5 has weak deacetylase activity but can catalyze the removal of three acidic lysine modifications, malonylation, succinylation, and glutarylation [21], and is the only desuccinylase located in the cytoplasm [22, 23]. In the mouse and rat retinas, Sirt5 is mainly expressed in the ganglion cell layer (GCL), inner plexiform layer (IPL) and retinal pigment epithelium (RPE) [24, 25], and its expression is low in diabetic retinas due to its NAD^+^-dependent activity [24, 26]. Moreover, Sirt5 can regulate autophagy in the liver and in tumor cells [27], and Sirt5-knockout mice are characterized by an abnormal retinal electroretinogram [26, 28] and mitochondrial autophagy defects [29], suggesting that Sirt5 may be involved in the protection of retinal nerve function in DR. In the present study, we investigated a potential neurodegenerative mechanism of DR and demonstrated that Sirt5-mediated desuccinylation of the OPTN K108 site protects RGCs from autophagic flux dysfunction in DR.

## Results

### Identification of the succinylation of OPTN K108 in vitreous humor from DR patients by MS/MS assay

**Fig. 1:**
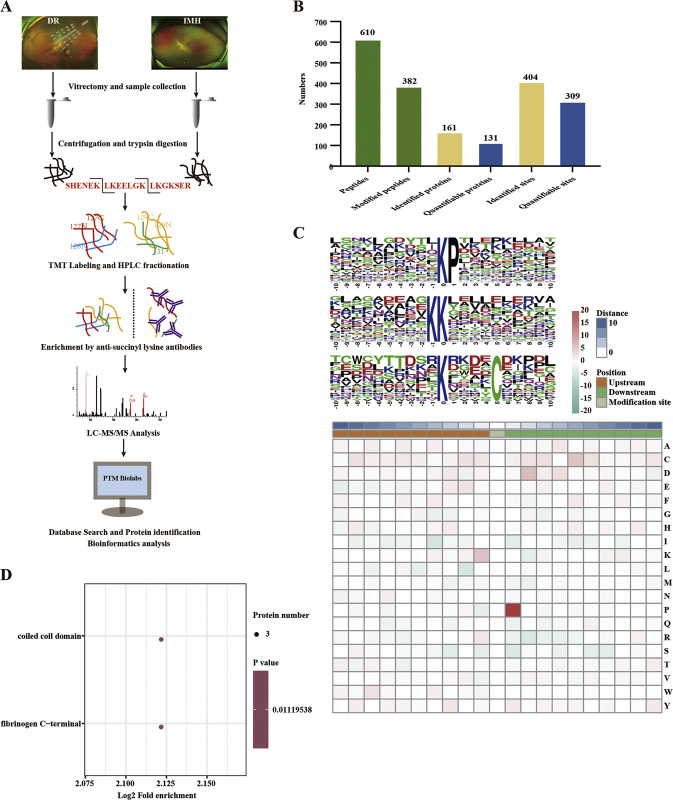
Lysine succinylome analysis of vitreous humor from DR and IMH patients. **A** Integrated strategy for the identification and quantitative profiling of the succinylome between the DR and IMH vitreous humor. **B** Basic statistical figure of MS results. **C** The motif enrichment heatmap of upstream and downstream amino acids of all identified modification sites. Red indicates that this amino acid is significantly enriched near the modification site, and green indicates that this amino acid is significantly reduced near the modification site. **D** Protein domain enrichment bubble plot of proteins corresponding to all identified modification proteins. DR diabetic retinopathy, IMH idiopathic macular hole, MS mass spectrum.

We combined TMT labeling, highly specific anti-succinyl lysine pan-antibody enrichment, and LC–MS/MS analysis for the systematic analysis of the succinylome in vitreous humor samples from patients with DR or idiopathic macular hole (IMH) (Fig. 1A). We identified 404 sites in 161 proteins that were modified by succinyl in vitreous humor, with 309 sites in 131 proteins containing quantitative information (Fig. 1B). Of the identified sites, most modification sites were located on three motifs, K^su^P, K^su^K, and K^su^xxxxC (Fig. 1C and Supplementary Table 1), and two domains, a coiled-coil domain and a fibrinogen C-terminal domain (Fig. 1D).

**Fig. 2:**
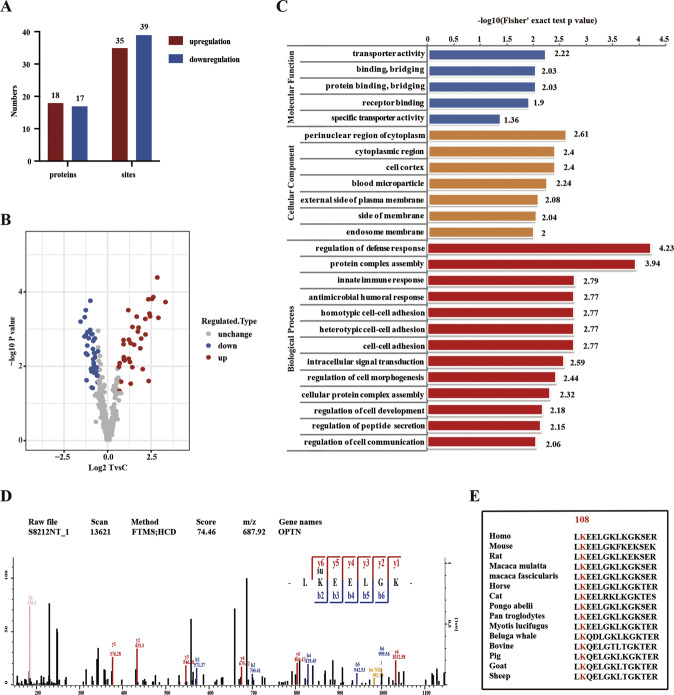
Identification of differentially succinylated proteins between DR and IMH. **A** The number of differentially succinylated proteins or modification sites between the DR and IMH groups. Quantitative data with a *p* value < 0.05 and ratio >1.5 or <0.667 were considered differential succinylation. **B** Volcano plot of differentially succinylated modification sites between the DR and IMH groups. **C** Histogram of GO enrichment analyses based on the cellular component, molecular function and biological process categories of differentially succinylated proteins. **D** MS spectrum of the succinylated OPTN K108 site. **E** The K108 site is highly conserved in different species, ranging from mice to humans. DR diabetic retinopathy, IMH idiopathic macular hole, GO Gene Ontology, K lysine.

Quantitative data with a *p* value < 0.05 and ratio >1.5 or <0.667 were considered differential succinylation, and a total of 74 sites in 35 proteins were differentially succinylated between DR and non-DR vitreous humor samples; of these sites, 35 sites in 18 proteins showed increased succinylation, and 39 sites in 17 proteins showed decreased succinylation (Fig. 2A, B). We next employed Gene Ontology (GO) enrichment analysis to analyze the role of differentially succinylated proteins in vitreous humor, which were mainly localized to the cytoplasm in cellular component analysis and enriched in transporter activity, protein binding and receptor binding ability according to molecular function analysis; analysis based on the biological process category showed that the differentially succinylated proteins were significantly enriched in regulation of the defense response (Fig. 2C). Among these proteins, we were particularly interested in OPTN, which is succinylated at a single lysine, Lys108 (K108^su^) (Fig. 2D), and highly conserved in different species, ranging from mice to humans (Fig. 2E).

### Verification of OPTN succinylation at the K108 site in the retina

**Fig. 3:**
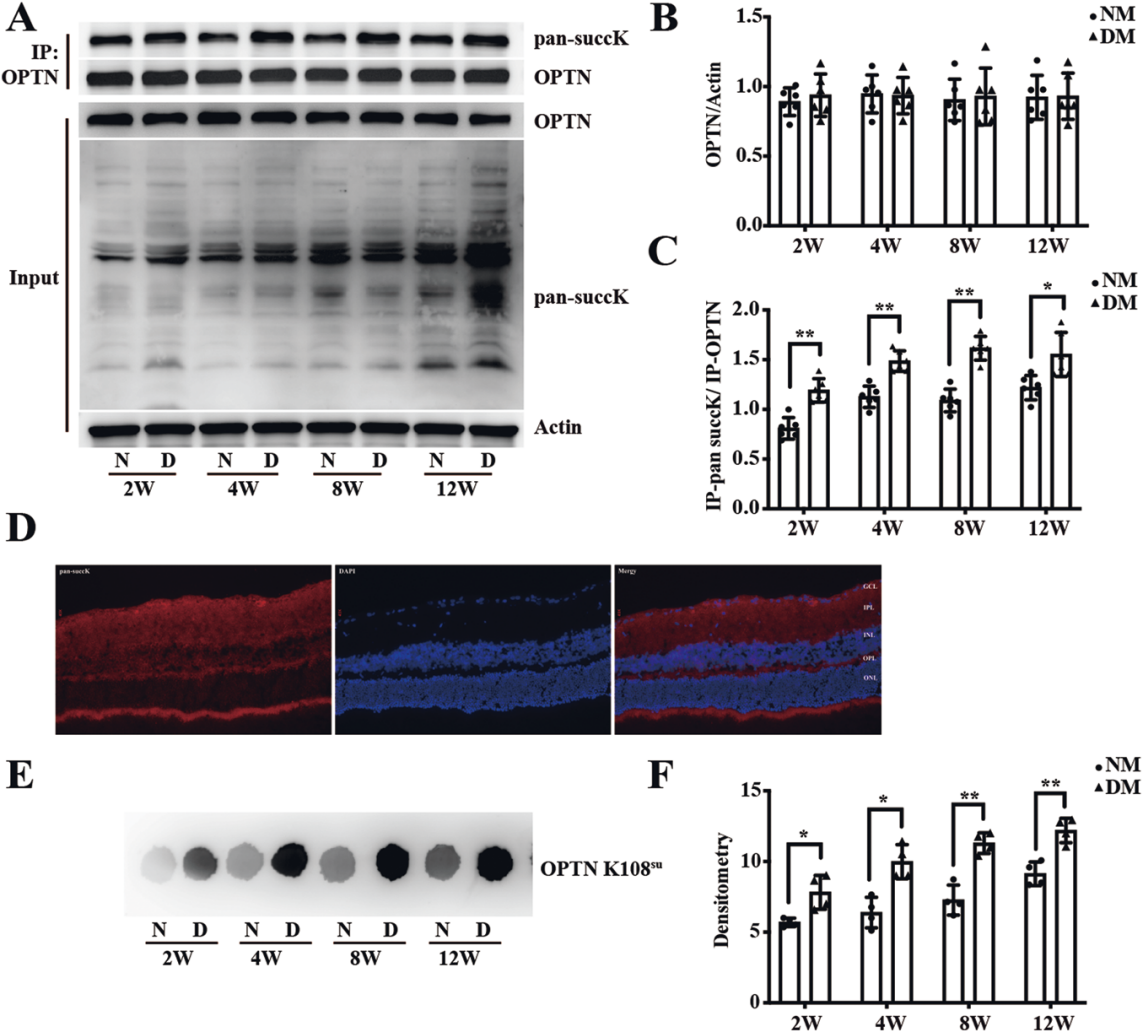
The expression of OPTN K108^su^ in the rat retina. **A** Endogenous OPTN was succinylated in rat retinas. OPTN was immunoprecipitated from whole retinal proteins using an anti-OPTN antibody, followed by western blotting with an anti-pan-succK antibody. Global protein succinylation and OPTN expression in whole retinal lysate were detected by western blotting. Bar graph showing the relative expression of OPTN protein (normalized to β-actin) in whole retinal lysate (**B**) and the pan-succinylation level of IP-OPTN (normalized to IP-OPTN, **C**). **D** Immunofluorescent staining of succinylation proteins in the retina. **E**, **F** Endogenous OPTN K108^su^ was succinylated in rat retinas. Succinylation of the OPTN K108 site was detected by dot blotting with a custom-made site-specific anti-succK108-OPTN antibody. Representative four immunoblots (**D**) and quantitative data (**E**) for each group are provided (*n* = 4). The data are shown as the mean ± SD (*n* = 6). *p < 0.05. **p < 0.01. OPTN K108^su^ succinylation of the OPTN lysine 108 site, IP immunoprecipitation, NM normal glucose group, DM diabetes mellitus group.

To confirm the succinylation of OPTN at the K108 site (OPTN K108^su^) in the retina, we established a streptozotocin (STZ)-induced T1DM Sprague–Dawley (SD) rat model and detected the expression of the corresponding protein at 2, 4, 8, and 12 weeks. Interestingly, we found no differences in OPTN expression in retinas between control retinas from normal glucose (NM) rats and rats with STZ-induced diabetes mellitus (DM) at 2, 4, 8, and 12 weeks (Fig. 3A, B); however, a time-dependent increase in the level of global succinylation was detected starting from 2 weeks after STZ treatment compared to the control treatments (Fig. 3A). Then, we assessed the succinylation of OPTN by immunoprecipitation (IP) using an anti-OPTN antibody followed by immunoblotting with an anti-pan-succK antibody. Our results showed that compared to that in the control retinas, endogenous OPTN in the diabetic rat retinas was heavily modified by succinylation at 2, 4, 8, and 12 weeks (Fig. 3A, C). The succK-modified proteins were concentrated in the ganglion cell layer (GCL), inner plexiform layer (IPL) (Fig. 3D). In addition, to further ascertain whether OPTN K108^su^ varied in the retinal tissues, we used an anti-OPTN succinyl-K108 specific antibody to detect the level of OPTN K108^su^, which was markedly increased in the diabetic retinas compared to the control retinas, as assessed by dot blots performed at 2, 4, 8, and 12 weeks (Fig. 3E, F). These observations suggested that OPTN was indeed succinylated at the K108 site in the DR retina.

### Lys108 in OPTN is a key site for high glucose-mediated succinylation and autophagic flux blockade in rRGCs and R28 cells

**Fig. 4:**
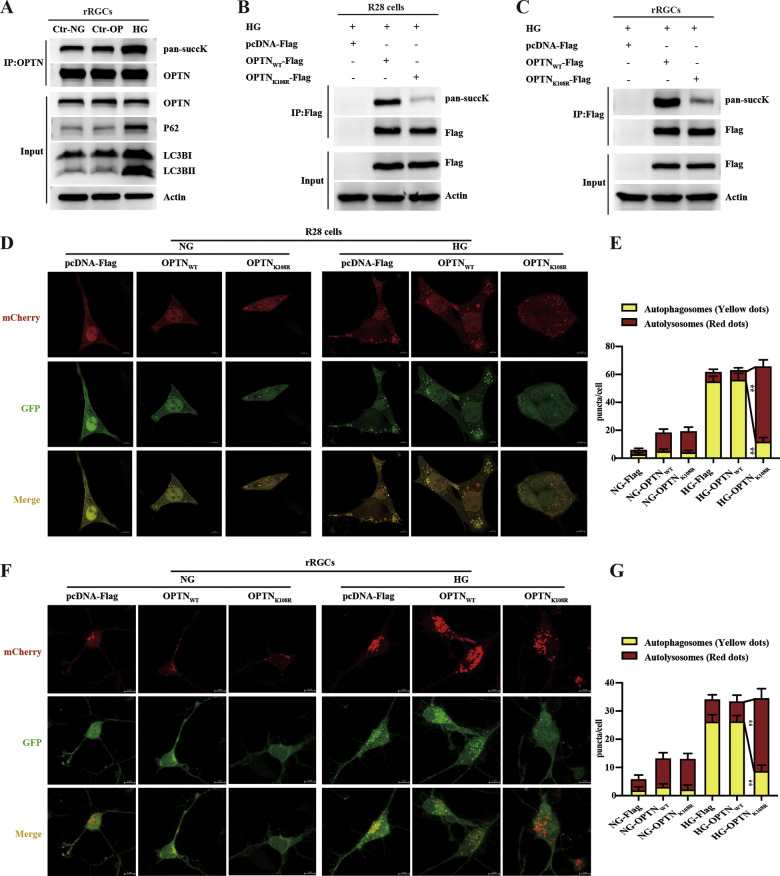
Desuccinylation of OPTN activates autophagic flux in R28 cells and rRGCs. **A** High glucose induced the upregulation of OPTN succinylation and the blockade of autophagic flux in primary rRGCs. The rRGCs were cultured under high-glucose conditions (25 mM D-glucose), 5.5 mM D-glucose as a control group, and 20 mM mannitol (plus 5.5 mM glucose) as a control for osmotic pressure (OP). The Flag tag was immunoprecipitated, followed by immunoblotting with an anti-pan-succK antibody. Whole-cell lysates were used as the input and analyzed by western blotting for the expression of OPTN and the autophagic markers LC3BII/I and P62. **B**, **C** The OPTN K108R mutant abolished the succinylation of OPTN in R28 cells and primary rRGCs. Cells were transfected with the indicated plasmids under high-glucose conditions (25 mM D-glucose), and the Flag tag was immunoprecipitated, followed by immunoblotting with an anti-pan-succK antibody. Whole-cell lysates were used as the input and analyzed by western blotting for the expression of Flag and Actin. **D**, **F** The OPTN K108R mutant activated autophagic flux in R28 cells and primary rRGCs. Representative images of primary rRGCs and R28 cells transfected with mCherry-GFP-LC3B lentivirus following transfection with the indicated plasmids under normal or high-glucose conditions (5.5 mM or 25 mM D-glucose). Scale bar = 5 μm. **E**, **G** The number of red (autolysosomes) and yellow puncta (autophagosomes) per cell was determined using ImageJ. Twenty cells from six independent experiments were analyzed in each group. The data are shown as the mean ± SD (*n* = 6). *p < 0.05, **p < 0.01. HG high glucose.

As endogenous OPTN was succinylated in the vitreous humor and the retina of DR, we next explored the modification of OPTN and the role of OPTN K108^su^ using in vitro experiments. To exclude high glucose-induced changes due to changes in osmotic pressure (OP), we used not only normal glucose (NG) as a control but also high mannitol as an osmotic pressure control [30]. There was no significant difference between the osmotic pressure group and the normal glucose group, which showed that high glucose(HG)-induced changes were not due to changes in osmotic pressure (Fig. 4A). OPTN was more modified by succinyl under high-glucose conditions compared to the control group under normal glucose and osmotic pressure; meanwhile, we evaluated autophagic flux changes by examining the autophagosome markers LC3BII/I and substrate p62/SQTSM1 (P62). Our results showed that the protein expression levels of LC3BII/I and P62 in high-glucose conditions were higher than those in the control group, which indicated autophagic flux blockade under high-glucose conditions (Fig. 4A). Then, the OPTN K108R mutant that mimics desuccinylation was transfected into R28 cells and rRGCs under high-glucose conditions with the wild-type control plasmid pcDNA(3.1)-OPTN_WT_-Flag and the scrambled plasmid pcDNA(3.1)-Flag. There was stable expression of Flag in the WT and K108R groups and no Flag expression in the scrambled group, which suggested that the WT and mutant plasmids were stably expressed in cells (Fig. 4B, C). Then, OPTN K108^su^ was detected by immunoprecipitating OPTN and immunoblotting with an anti-pan-succK antibody. Wild-type OPTN was succinylated under high-glucose conditions, and the K108R mutant was succinylated at barely detectable levels in both R28 and primary rRGC cells (Fig. 4B, C), which suggested that OPTN K108R could abolish the succinylation of OPTN in retinal nerve cells.

Next, we examined autophagic flux using an mCherry-GFP-LC3B reporter lentivirus. There were few red and yellow puncta under normal-glucose conditions, indicating reduced levels of autophagy and no blockade of autophagic flux. More red and yellow puncta were induced under high-glucose conditions than under normal-glucose conditions, which indicated an induction of autophagy and a blockade of autophagic flux under high-glucose conditions. Under normal glucose conditions, autophagic flux was not blocked, and at a low level, overexpression of OPTN_WT_ or OPTN_K108R_ induced no obvious difference in yellow puncta. Under high-glucose conditions, there were fewer yellow puncta in the OPTN_K108R_-Flag-transfection group than in the WT group (Fig. 4D–G), which suggested that desuccinylation of the OPTN K108 site (desuccK108-OPTN) could restore high glucose-induced autophagic flux blockade in both R28 and primary rRGC cells. All of the above results suggested that the succinylation of OPTN at the K108 site under high-glucose conditions may be involved in high glucose-induced autophagic flux blockade.

### Sirt5 ameliorates high glucose-induced autophagic flux blockade by desuccinylating OPTN K108^su^

**Fig. 5:**
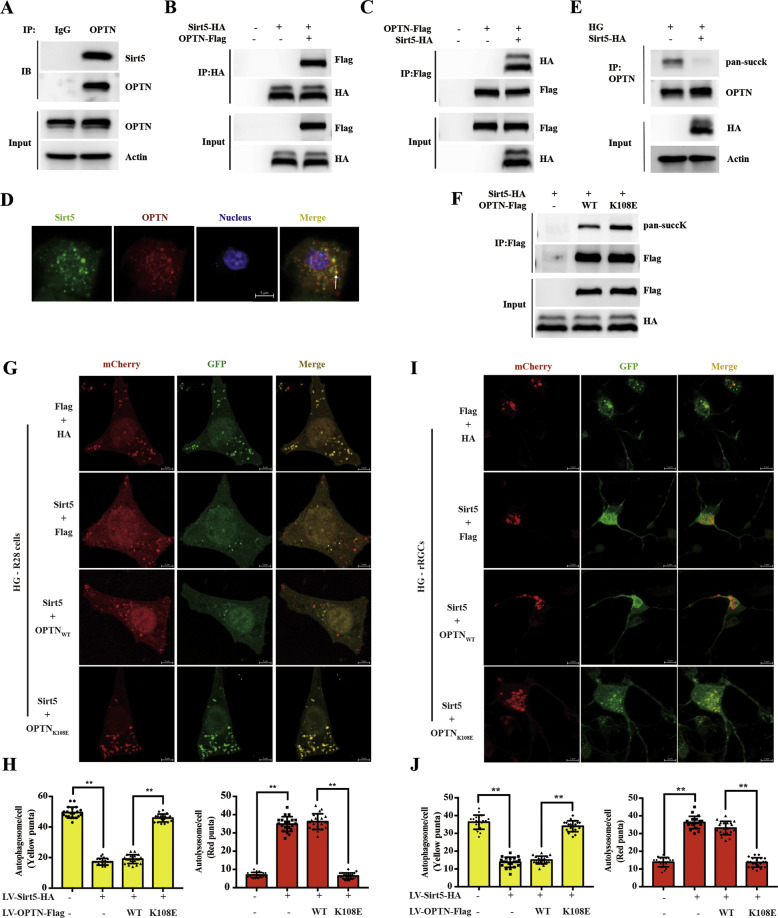
Sirt5-dependent desuccinylation of OPTN promotes autophagic flux. **A** Endogenous OPTN bound to endogenous Sirt5 in 293T cells. Endogenous Co-IP of OPTN and Sirt5 was performed as indicated in the lysates of 293T cells, and the association was examined by western blotting. **B, C** Tagged Sirt5 interacted with and tagged OPTN. 293T cells were transfected with the indicated plasmids, and the OPTN-Sirt5 association was examined by Co-IP and western blotting. **D** Exogenous OPTN colocalized with Sirt5 in 293T cells. 293T cells were transfected with lentiviruses encoding OPTN-Cherry and Sirt5-Zsgreen. Scale bar = 5 μm. **E** Sirt5 desuccinylated endogenous OPTN. 293T cells were transfected with the Sirt5-HA plasmid, followed by IP-OPTN. The succinylation level of OPTN was determined by western blotting. **F** The OPTN K108E mutant abolished the interaction with Sirt5. 293T cells overexpressing Sirt5 were transfected with the indicated plasmids, and their interaction with OPTN was analyzed by IP and western blotting. **G**, **I** The OPTN K108E mutant abolished Sirt5-induced autophagic flux activation of OPTN in R28 cells and primary rRGCs. Representative images of primary rRGCs and R28 cells transfected with mCherry-GFP-LC3B lentivirus following transfection with the indicated plasmids under high-glucose conditions (25 mM D-glucose). Scale bar = 5 μm. **H**, **J** The number of red (autolysosome) and yellow puncta (autophagosome) per cell was determined using ImageJ. Twenty cells from six independent experiments were analyzed in each group. The data are shown as the mean ± SD (*n* = 6). *p < 0.05. **p < 0.01. 293T cells human embryonic kidney 293T, Co-IP coimmunoprecipitation, IP immunoprecipitation, K108E mutant Lys-to-Glu (K-to-E) substitution to mimic succinylation overexpression.

To investigate whether Sirt5 could desuccinylate OPTN, we first verified that OPTN could interact with Sirt5. Coimmunoprecipitation and immunoblotting in 293T cells showed that endogenous OPTN interacted with Sirt5 (Fig. 5A). The interaction between exogenous pcDNA-OPTN-Flag and exogenous pcDNA-Sirt5-HA was also readily detected in 293T cells by coimmunoprecipitation and immunoblotting (Fig. 5B, C). Then, 293T cells were transfected with lentivirus encoding OPTN-Cherry and Sirt5-Zsgreen, and OPTN colocalized with Sirt5 in 293T cells (Fig. 5D). Second, to investigate the function of Sirt5 in OPTN desuccinylation, we overexpressed Sirt5-HA under high-glucose conditions and detected the levels of endogenous OPTN succinylation in 293T cells. As shown in Fig. 5E, overexpression of Sirt5 reduced the succinylation of endogenous OPTN. To further demonstrate that Sirt5-induced desuccK-OPTN at the K108 site, we generated OPTN K108E mutants to mimic oversuccinylation. Compared to the Sirt5+OPTN_WT_ group, the OPTN K108E mutant abolished the Sirt5-mediated desuccinylation of OPTN (Fig. 5F). Together, these results indicated that Sirt5 desuccinylated OPTN at its K108 site.

Third, we continued to explore the effect of Sirt5 on autophagic flux. As shown in Supplementary Fig. 1, the plasmid encoding OPTN_WT_-Flag or the OPTN_K108E_-Flag mutant with Sirt5-HA was stably expressed. The Sirt5-OPTN-mediated autophagic flux in R28 cells and primary rRGCs was assessed by the mCherry-GFP-LC3B lentivirus. The number of yellow puncta decreased significantly in the Sirt5-treated group compared to the control group; however, pretreatment with Sirt5 + OPTN_K108E_ followed by high glucose increased the number of yellow puncta and decreased the number of red puncta (Fig. 5G–J). Therefore, desuccK108-OPTN was critical for Sirt5 to reverse high glucose -induced autophagic flux dysfunction.

### The Sirt5-OPTN K108 axis promotes the formation of autolysosomes and protects RGCs function in STZ-induced diabetic rats

**Fig. 6:**
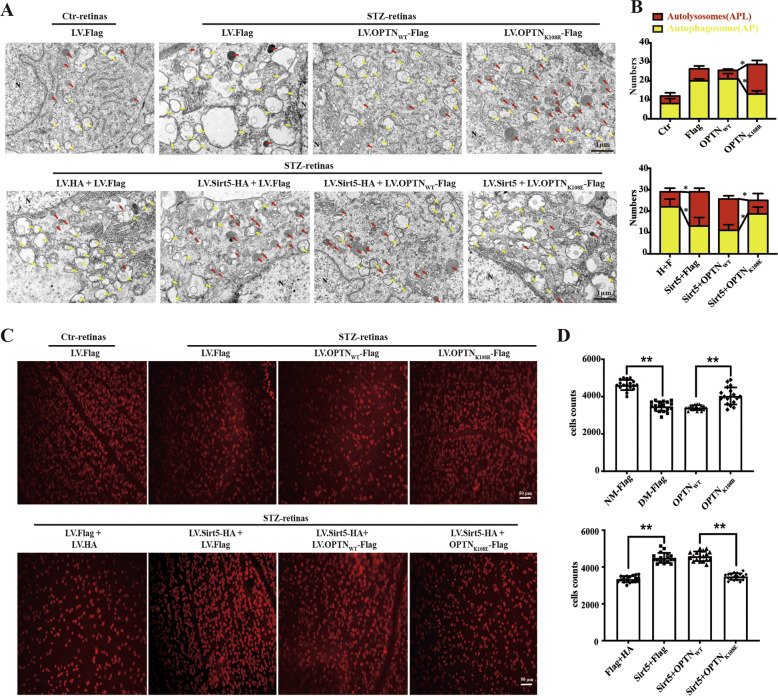
The Sirt5-OPTN K108^su^ axis induces the formation of autolysosomes and alleviates RGCs loss in STZ-induced diabetic rats. **A**, **B** TEM images of RGCs from eyes injected with the indicated lentivirus. Note the accumulation of AP in diabetic retinas. The presence of Sirt5 and the K108R mutant improved APL degradation. AP is indicated by yellow arrows, and APL is indicated by red arrows. Representative images (**A**) and quantitative data (**B**) for each group are provided. Scale bars: 1 µm. **C**, **D** Quantification of RGCs in rat retinal whole mounts. The phenotypic marker RBPMS labeled the soma of RGCs. Representative images (**C**) and quantitative data (**D**) of RBPMS^+^ RGCs subjected to eye injection of the indicated lentivirus. The data are shown as the mean ± SD (*n* = 6). *p < 0.05. **p < 0.01. TEM transmission electron microscopy, AP autophagosome, APL autophagolysosome, NM normal glucose, DM diabetes mellitus.

Subsequently, we performed in vivo experiments using an STZ-induced T1DM rat model to further investigate the potential neuroprotective effect of the Sirt5-OPTN K108 axis. The lentivirus (LV) was injected into the vitreous chamber of rats with STZ-induced diabetes at 1 week. As shown in Supplementary Fig. 2, the retina stably expressed Sirt5-HA, OPTN-Flag or mutant forms of the OPTN protein at 8 weeks after the injection of STZ. The change in autophagic process in each group was assessed by retinal TEM. Double parallel membrane layers of autophagosomes (APs) were partially visible through TEM, and autolysosomes (APLs) with one limiting membrane were identified by electron-dense cytoplasmic material and organelles at various stages of degradation. The injection of LV.OPTN_K108R_ increased the number of APLs compared to that in the LV.OPTN_WT_ group, and LV.Sirt5-HA + LV.OPTN_K108E_ could abolish the LV.Sirt5-induced AP decrease and APL increase (Fig. 6A, B). These findings suggested the above speculation that desuccinylation of OPTN K108 promoted the formation of autolysosomes. Immunohistochemical analysis using RBMPS phenotypic markers of RGCs was performed in whole retinal mounts to assess RGCs loss. RGCs number after the injection of LV.OPTN_K108R_ was significantly increased compared to that of the LV.OPTN_WT_ group. LV.Sirt5 increased the RGCs numbers compared to control DM retinas, but the LV.OPTN_K108E_ reversed this effect compared to the Sirt5+LV.OPTN_WT_ group (Fig. 6C, D).

**Fig. 7:**
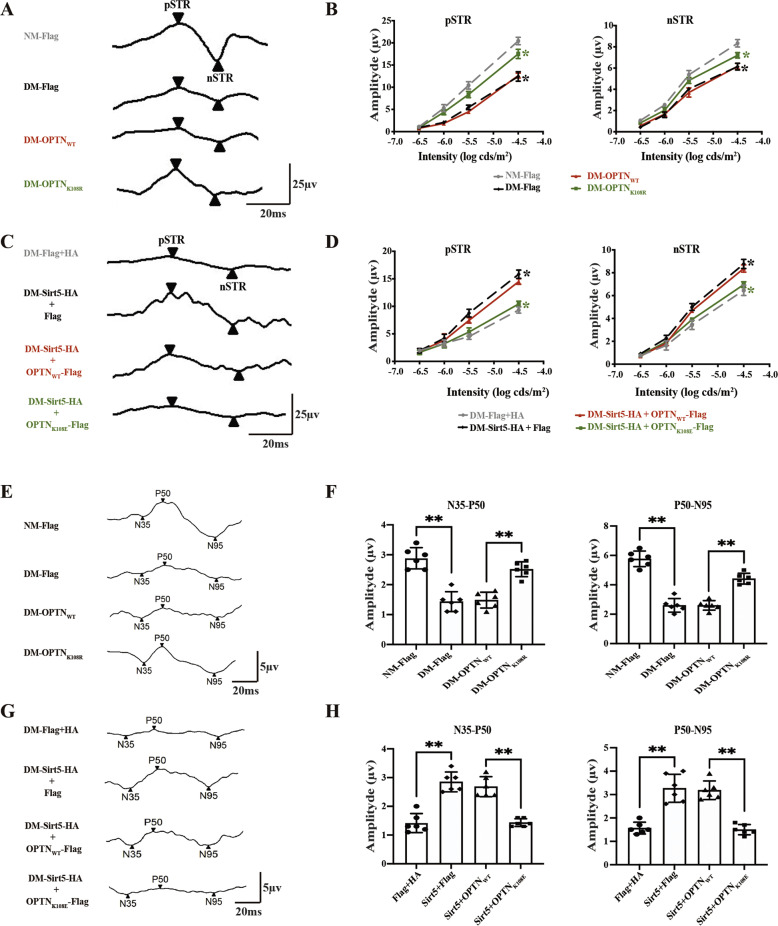
The Sirt5-OPTN K108^su^ axis improves RGCs function in STZ-induced diabetic rats. **A**, **B** Stimulus intensity responses for the STR, showing the pSTR and nSTR in rats whose eyes were injected with OPTN-WT, OPTN-K108R or scrambled lentivirus. Representative images (**A**) and quantitative data (**B**) are provided. Absolute amplitude appears on the y-axis, and the 4 flash intensities (log cds/m^2^) are on the x-axis. A description of the data points appears at the bottom of the graph. Green *p < 0.05 between LV.OPTN_K108R_- and LV.OPTN_WT_-injected groups; black *p < 0.05 between DM and NM retinas subjected to LV.Flag. **C**, **D** pSTR and nSTR in rats subjected to eye injection of LV.Sirt5 and OPTN-WT, OPTN-K108E or scrambled lentivirus. Representative images (**C**) and quantitative data (**D**) are provided. Green* indicates *p* < 0.05 between the LV.Sirt5 + OPTN_K108E_- and LV.Sirt5 + OPTN_WT_-injected groups. Black* indicates *p* < 0.05 between the LV-Sirt5+Flag and scrambled LV.HA + Flag groups. **E**, **F** PERG in rats subjected to eye injection of OPTN-WT, OPTN-K108R or scrambled lentivirus. Representative images (**E**) and quantitative data (**F**) are provided. **G**, **H** PERG in rats subjected to eye injection of LV.Sirt5 and OPTN-WT, OPTN-K108E or scrambled lentivirus. Representative images (**G**) and quantitative data (**H**) are provided. The data are shown as the mean ± SD (*n* = 6). *p < 0.05. **p < 0.01. STR scotopic threshold response, pSTR/nSTR positive STR and negative STR, PERG pattern electroretinogram.

We proceeded to evaluate the impact of the Sirt5-OPTN K108^su^ axis on RGCs function using electrophysiological techniques. The STR amplitude of LV-OPTN_K108R_ showed a moderate increase compared to that of LV.OPTN_WT_ (Fig. 7A, B). The increase in STR amplitude with LV-Sirt5 was ablated by LV.OPTN_K108E_ (Fig. 7C, D). Similar results were obtained by analyzing the amplitude of PERG (Fig. 7E, H). These results highlighted the possibility that RGC protection mediated by the Sirt5-OPTN functional pair might be largely mediated by Sirt5 desuccinylation of OPTN K108^su^.

## Discussion

This study aimed to investigate and compare the retinal succinylome in DR and identify the key factor that plays a vital role in this process. To our knowledge, this study is the first systematic analysis characterizing the intravitreal succinylome of DR eyes. Lysine succinylation was identified as a new posttranslational modification in 2011, and the succinyl-CoA and sodium succinate formed through the tricarboxylic acid cycle (TCA) cycle significantly enhanced the global profile of succinylation [7]. Many studies have shown that hyperglycemia impairing the TCA cycle leads to the generation of more lysine-succinylated proteins [8], and a study on wild-type *E. coli* showed that high-glucose conditions increased the abundance of succinyllysine peptides more significantly than acetylation peptides [31]. The upregulation of succinylation modification in the diabetic vitreous humor and retina observed in our research is supported by past studies demonstrating an accumulation of succinate caused by hyperglycemia in DR [6, 32, 33], which provides a prerequisite to the occurrence of succinylation modification. Moreover, Sirt5 expression is low in diabetic rat retinas due to its NAD^+^-dependent activity [24, 26], resulting in decreased desuccinylase activity, which also supports the increase in protein succinylation modification in diabetic retinas.

The changes in single protein succinylation modification are generally due to one of two possibilities: one possibility is that the protein level itself remains unchanged, while the modification level changes; the other possibility is that the protein level itself changes, and the changes in modification are a reflection of the changes in the global protein level. We reported here that the overall expression level of OPTN showed no differences, while the succinylation of OPTN was upregulated between diabetic retinas and nondiabetic retinas. Thus, the succinylation of OPTN, not the protein level of OPTN, is involved in DR. DR is a progressive disease characterized by early neuronal dysfunction and late vascular abnormalities [2]. We showed here that the succinylation of OPTN was fixed, while a time-dependent increase in the level of global succinylation was detected during STZ-induced T1DM. We suggest that the succinylation of OPTN may occur in early diabetes, be involved in RGCs dysfunction, and remain stable during the course of diabetes. However, as diabetes progresses, an increasing number of proteins modified by succinyl increase pan-succinylation accumulation, which drives pathological changes.

Studies on OPTN in eye disease have mainly focused on primary open-angle glaucoma (POAG), which is a group of inherited disorders. POAG is characterized by the loss of RGCs and results from the presence of mutations in specific genes [34] and mutations in OPTN, including the E50K, M98K, H486R, R545Q, H26D, E322K, E103D, and V148V sites [14]. In-depth data showed that the E50K and M98K mutations of OPTN were involved in the autophagic process and caused the death of RGCs in POAG [13]. Autophagy is also an early event in DR, and early studies found that the autophagy machinery is activated by the beclin-1 signaling pathway under high-glucose conditions in DR, producing higher amounts of autophagosomes in the cytosol, with an upregulation of LC3BII/I [35, 36]. Recent studies showed that an accumulation of p62/SQTSM1 cargo occurs when the autophagic flux is compromised and then induces neurodegeneration in DR [19, 37], which echoes the finding that the autophagic flux is disrupted in other neurodegenerative disorders, such as Parkinson’s disease [38]. In this study, we suggest that OPTN K108^su^, caused by hyperglycemia-induced succinate accumulation, disrupts autophagic flux in RGCs, thus resulting in the accumulation of oxidatively damaged proteins or organelles in the cytoplasm, which might mechanistically underlie RGCs damage.

To study RGCs function, we used not only primary cultured rat RGCs but also the retinal precursor cell line R28. R28 cells originated from a postnatal day 6 rat retinal culture and are used for a variety of studies of retinal cell behavior, including differentiation, neuroprotection, cytotoxicity, and light stimulation, as well as retinal gene expression and neuronal function [39]; we used these cells to strengthen the findings obtained from primary rRGCs culture. Finally, we also used an STZ-induced diabetes rat model with intravitreal injection of lentiviral particles to validate the role of OPTN in RGCs loss and function in vivo. The eyeball is a relatively immune-privileged area that can avoid the immune response caused by viral vectors. Previous studies also provided evidence that an lentiviral vector could be stably expressed in the rat inner retina after intravitreal injection [6]. The phenotypic marker RBPMS labels the soma of RGCs, which is currently considered the most appropriate RGCs marker due to providing an accurate measure of the entire RGCs population [40]. As surviving RGCs are not necessarily functional, we used STR and PERG to obtain a waveform that is ganglion cell dependent.

In particular, our findings first showed the desuccinylation function of Sirt5 for OPTN K108^su^ in 293T cells; this result might thus be of broad significance across cell types. Moreover, we reported that Sirt5-mediated autophagic flux activation by desuccinylating OPTN K108^su^, which echoed past reports that Sirt5-induced deacetylation of lactate dehydrogenase B (LDHB) at the K329 site triggered hyperactivation of autophagy and colorectal cancer cell growth [41] and that Sirt5 could promote autophagic flux by affecting both the early and late stages of the autophagy process in osteosarcoma cells exposed to DNA damage [42]. In addition, past studies have found that Sirt5 germline-knockout (Sirt5^−/−^) mice exhibited modest retinal dysfunction in terms of the amplitudes of electroretinography (ERG) and showed neuroprotective effects in the inner retina under hyperglycaemic conditions [28]. Sirt3 and Sirt5 double-knockout mice (Sirt3^KO^ Sirt5^KO^) were strikingly more vulnerable to retinal degeneration upon light stress, as assessed by ERG, revealing important roles in photoreceptor survival. Consistent with these findings, we reported here that the overexpression of Sirt5 could decrease RGCs loss and protect RGCs function, as assessed by desuccinylating OPTN K108^su^ in rats with STZ-induced diabetes.

This study has some limitations. First, this study used human vitreous humor samples from idiopatic macular hole patients as non-DR controls. Because samples from healthy people were unavailable, we used vitreous humor samples from idiopatic macular hole patients who required vitrectomy treatment, which is the most commonly used approach for human retinal proteomic research on vitreoretinal eye diseases [43, 44]. Second, OPTN is a cytoplasmic rather than a secretory protein that is preferentially expressed in RGCs;[12] it should logically not be found in the aqueous humor, but we indeed detected it by LC–MS/MS analysis, with a T/C ratio of 1.53 in human DR vitreous humor compared to non-DR vitreous humor. We infer that cell death of retinal cells may result in exocytosis of the OPTN protein, but the mechanism remains to be explored. Moreover, considering that OPTN forms a complex with p62/SQTSM1 and facilitates its autophagic degradation [17], p62/SQTSM1 was not suitable for determining pan-autophagic flux in our case; thus, the change in autophagic flux was not assessed by substrate changes. Finally, due to the limited specificity of the anti-OPTN-succinyl-K108 antibody, we could only perform dot blot assays in place of western blot assays.

In conclusion, we have demonstrated that the succinylome of the vitreous humor in DR is significantly enriched in the regulation of the defense response, and OPTN K108^su^ occurs in the rat retina during DR and mediates autophagic flux blockade under high-glucose conditions. Sirt5 desuccinylated OPTN K108^su^, thus protecting RGCs function from high glucose-induced RGCs autophagic flux blockade in the DR retina. In particular, with the strong association between RGCs autophagic flux dysfunction and Sirt5 deficiency or OPTN succinylation in the retina, targeting the Sirt5-desuccK108-OPTN axis may thus open a new avenue for therapeutic intervention in DR neurodegeneration.

## Materials and methods

### Vitreous humor (VH) collection

Human DR VH (*n* = 15) and matched VH from nondiabetic idiopatic macular hole (IMH) patients (*n* = 15) were obtained from vitrectomy specimens at the Department of Ophthalmology, Shanghai Jiao Tong University Affiliated Sixth People’s Hospital, from April 2017 to March 2018. To exclude individual differences, we randomly pooled sets of five samples from each group together and obtained 3 mixed samples for the DR group and 3 mixed samples for the non-DR group. This study was approved by the Research Ethics Board of Affiliated Shanghai Sixth People’s Hospital and was conducted according to the World Medical Association Declaration of Helsinki. Signed informed consent was obtained from all subjects before inclusion in the protocol.

### Trypsin digestion, TMT labeling, high-performance liquid chromatography (HPLC) fractionation, Ksu peptide affinity enrichment, liquid chromatography–tandem mass spectrometry analysis and database searching, and bioinformatics analysis

Human vitreous humor pretreatment and TMT analysis were performed as previously described [45, 46]. Peptide was reconstituted in 0.5 M TEAB, processed according to the manufacturer’s protocol for the TMT kit/iTRAQ kit, and fractionated by high pH reverse-phase HPLC using a Thermo Betasil C18 column (5-μm particles, 10 mm ID, 250 mm length). To enrich succinylation-modified peptides, tryptic peptides dissolved in NETN buffer (100 mM NaCl, 1 mM EDTA, 50 mM Tris-HCl, 0.5% NP-40, pH 8.0) were incubated with prewashed anti-pan-succinylation beads (PTM Bio, PTM-402) at 4 °C overnight with gentle shaking. For LC–MS/MS analysis, the resulting peptides were desalted with C18 ZipTips (Millipore, USA) according to the manufacturer’s instructions. The peptides were subjected to NSI source, followed by tandem mass spectrometry (MS/MS) on a Q ExactiveTM Plus (Thermo Fisher, USA) coupled online to UPLC. The electrospray voltage applied was 2.0 kV. The m/z scan range was 350–1800 for the full scan, and intact peptides were detected in the Orbitrap at a resolution of 70,000. The resulting MS/MS data were processed using the MaxQuant search engine (v.1.5.2.8). Gene Ontology (GO) analysis was performed using the Database for Annotation, Visualization and Integrated Discovery (DAVID) against the background of Homo sapiens.

### Development of the rabbit anti-OPTN-succinyl-K108 antibody

The antibody specifically identifying succinyl at the OPTN K108 site was developed by PTM Bio. (Chicago, IL, USA). Briefly, two succinyl-modified peptides and one nonmodified control peptide (Supplementary Table 2) were designed and synthesized. After MS detection, 3 rabbits were immunosuppressed via injection of two succinyl-modified peptides coupled with KLH. Then, serum screening by ELISA and dot blotting with positive results were performed for purification. Antibodies purified from 3 rabbits were named Ab1/2/3, and Ab1 was discarded. The purified Ab2/3 antibody was verified by ELISA (Supplementary Table 3) and dot blotting (Supplementary Fig. 3).

### STZ-induced diabetes rat model

Male Sprague–Dawley rats (200 ± 10 g, six per experimental group) were obtained from the Animal Laboratory of Shanghai Jiao Tong University Affiliated Sixth People’s Hospital. All animal interventions were approved by the Animal Ethics Committee of Shanghai Jiao Tong University Affiliated Sixth People’s Hospital and were processed in accordance with the guidelines of the Animal Care and Use Committee of the National Institutes of Health. Rats were randomly separated into the control or DM groups. After 12 h of fasting, the DM groups were given an intraperitoneal injection of 60 mg/kg STZ, and the same dose of citric acid buffer was injected intraperitoneally into the control group. Fasting blood samples were taken from the tail vein of rats, the blood glucose levels of all rats were tested after 48 h of STZ injection, and a fasting blood glucose >16.7 mmol/L represented successful establishment of the diabetic rat model.

### R28 cells, 293T cells and primary rat RGCs culture

The R28 rat retinal precursor cell line and human embryonic kidney 293T (293T) cells were cultured in Dulbecco’s modified Eagle’s medium (DMEM) supplemented with 10% fetal bovine serum (FBS).

Primary rat retinal ganglion cells (rRGCs) cultures were prepared from day 1–3 SD rat retinas, as described in our previous reports on DR [47, 48]. Briefly, the retinas were isolated, digested in 5 mg/mL papain dissociation solution (Worthington, LS003126) for 20 min, and dispersed in fresh ovomucoid solution (Sigma Aldrich, USA). To purify the rRGCs, the cell suspension was incubated for 2 h at 37 °C on a second panning plate coated with sheep anti-mouse IgG (H + L) antibody (1:50, Jackson ImmunoResearch, 115-035-003) and anti-mouse Thy 1.1 antibody (1:50, Abcam, ab225) for 2 h at 37 °C. Adherent cells were digested from the plate by incubation with 0.125% trypsin for 5 min at 37 °C. The cells were cultured in serum-free Neurobasal^TM^-A Medium (Gibco, 10888022) with 2% B27, 1% progesterone, 1% putrescine, 1% sodium pyruvate, 1% T3 and T4 hormone, 1% insulin-transferrin-selenium (ITS, Biogems, 00-102), 1% BDNF (PeproTech, 071961), CNTF (PeproTech, 020865-1) and FGF (PeproTech, 1210432-1), 2 mM L-glutamine, 20 mg L-cysteine, 5 mg BSA, and penicillin/streptomycin. The cells were plated onto poly-D-lysine-coated glass-bottom cell culture dishes. The RGC was identified as our previous report [47, 48].

The R28 cell line and primary rRGCs exposed to 25 mM D-glucose were used to simulate a hyperglycaemic environment in vitro; 5.5 mM D-glucose was used as the control group, and 20 mM mannitol (plus 5.5 mM glucose) was used as a control for osmotic pressure.

### Plasmid construction and transfection

We carried out mutagenesis experiments as previously reported [7, 49]. OPTN mutants bearing Lys-to-Arg (K-to-R) substitutions at the succinylated K108 site (OPTN K108R) were generated to mimic desuccinylation, and Lys-to-Glu (K-to-E) substitutions were generated to mimic succinylation overexpression. Wild-type OPTN or the mutant vectors pcDNA3.1(+)-OPTN_WT_-Flag, pcDNA3.1(+)-OPTN_K108R_-Flag, pcDNA3.1(+)-OPTN_K108E_-Flag, and pcDNA3.1(+)-Sirt5-HA were constructed by Obiosh Biotechnology (Shanghai, China). The control plasmid contained only a Flag or HA label (pcDNA3.1(+)-Flag or pcDNA3.1(+)-HA). Transient transfection was performed using high-throughput compatible Lipofectamine™ LTX Reagent with PLUS™ Reagent (Thermo Fisher, 15338-100). Cells were harvested 48 h after transfection.

### Monitoring autophagic flux using the mCherry-GFP-LC3B lentivirus

R28 cells or rRGCs were seeded onto 35-mm glass-bottom dishes for confocal microscopy and transfected with OPTN or mutant plasmids. Six hours later, the medium was replaced with regular culture medium plus LV-mCherry-GFP-LC3B lentivirus (multiplicity of infection: 30, Obiosh Biotechnology, Shanghai, China). After 6 h, high-glucose medium (25 mM) was added, and the cells were cultured for an additional 48 h. Finally, the cells were stained with DAPI for 5 min in the dark. Images were captured using a fluorescence confocal microscope (Zeiss LSM 880, Jena, Germany). mCherry fluorescence is detectable in autophagosomes and in autolysosomes, but GFP fluorescence is seen only in autophagosomes and not in autolysosomes due to the sensitivity of GFP to the low pH in autolysosomes. Therefore, yellow puncta, reflective of the combination of red mCherry and green GFP fluorescence, indicate autophagosomes, whereas red puncta (mCherry only) indicate autolysosomes. Quantification of yellow or red puncta was performed as previously reported [50].

### Co-Immunoprecipitation (Co-IP)

IP was performed according to the instructions of the Pierce™ Crosslink Magnetic IP/Co-IP Kit (Thermo Fisher, 88805). Briefly, the beads were prewashed twice with 1X Modified Coupling Buffer in the kit and then bound to 5 μg of rabbit anti-IgG (Abcam, ab172730), rabbit anti-OPTN (Abcam, ab213556), rabbit anti-HA-Tag (CST, #3724) or rabbit anti-Flag (DYKDDDDK)-Tag (CST, #14793) antibody for 15 min, and the antibody was crosslinked to the beads with DSS for 30 min at room temperature. The beads were washed three times with elution buffer followed by two washes with IP lysis/wash buffer. The cell and retinal lysates were prepared using IP lysis buffer in the kit, and then the cell or tissue lysate was incubated with antibody-crosslinked beads overnight at 4 °C. The beads were washed twice with IP lysis/wash buffer and once with ultrapure water, and then the bound antigen was eluted. To neutralize the low pH, 10 μL of neutralization buffer was added to each 100 μL of eluate. The antigen eluate and the whole lysates were evaluated by western blot analysis using relevant antibodies and mouse anti-rabbit IgG (conformation-specific) antibody (CST, #3678), which did not recognize denatured and reduced IgG heavy or light chains.

### Western blot assay

Proteins were extracted from cultured cells and retinal tissues using radioimmunoprecipitation assay (RIPA) buffer and separated on 12.5% SDS-polyacrylamide gels. Proteins were probed with the following antibodies: rabbit anti-pan-succK (1:1000; PTM Bio, PTM-401); rabbit anti-OPTN (1:1000, Abcam, ab213556); rabbit anti-Sirt5 (1:1000, Abcam, ab78982); rabbit anti-LC3B (1:2000; Abcam, ab192890); rabbit anti-P62 (1:1000; CST, #23214); rabbit anti-HA-Tag (1:1000, CST, #3724); rabbit anti-Flag (DYKDDDDK)-Tag (1:1000, CST, #14793); and mouse anti-β actin (1:1,000, Beyotime, AA128) as an internal control. The secondary antibodies were HRP-conjugated goat anti-rabbit (1:5000, Jackson ImmunoResearch, 111-035-003) and HRP-conjugated goat anti-mouse (1:5000, Jackson ImmunoResearch, 115-035-003) antibodies. The blots were scanned with an Odyssey imager (LI-COR Biosciences, NE, USA), and the band intensity was determined using the Quantity One System (Bio–Rad, CA, USA).

### Dot blotting

Dot blot immunoassays were carried out on a PVDF membrane. Retinal lysate (10 μg) was dropped onto a PVDF membrane. After drying, the membrane was incubated with 5% BSA for 60 min, and the anti-OPTN-succinyl-K108 rabbit antibody (1:1000, PTM Bio, CTM241) and goat anti-rabbit IgG (H + L) (1:5000, Jackson ImmunoResearch, 111-035-003) were added. The blots were developed with an ECL chemiluminescence kit (Thermo Fisher, 34580).

### Lentivirus packaging and intravitreal injection

Sirt5 and WT or mutant OPTN were inserted into the GV367 lentivirus expression vector by Shanghai GeneChem Co., Ltd. (Shanghai, China), as previously described [51]. Then, rats with STZ-induced diabetes were randomly divided into eight groups, and each group received an intravitreal injection with a 30-gauge needle and a syringe (Hamilton, 7632-01). For the present study, rats with STZ-induced diabetes received intravitreal injections of 1 μl containing 2 × 10^8^ TU/mL LV particles: LV-Flag, LV-OPTN_WT_-Flag, LV-OPTN_K108R_-Flag, LV-HA and LV-Flag, LV-Sirt5-HA and LV-Flag, LV-Sirt5-HA and LV-OPTN_WT_-Flag, and LV-Sirt5-HA and LV-OPTN_K108E_-Flag. The LV of each group was injected into the vitreous chamber of rats with STZ-induced diabetes at 1 week. Given that RGC loss started at 4-6 weeks after DM induction in an animal model according to a previous report [52–54], we detected autophagic process and RGC changes at 8 weeks after the injection of STZ.

### Transmission electron microscopy (TEM) observation

The TEM was performed as previously reported [55]. The eyeballs of each group were collected and immediately placed in 2.5% glutaraldehyde (pH 7.2, Servicebio, G1102) for 30 min at 4 °C. The retinas were detached under an operating microscope, fixed for an extra 2 h at 4 °C, postfixed for 1 h with 1% osmium tetroxide (Ted Pella Inc., USA) at room temperature, and dehydrated via an ascending series of ethanol concentrations (maximum concentration of 100%). The retinas were embedded in EMBed 812 (SPI, 90529-77-4) and moved into a 65 °C oven to polymerize for more than 48 h. The resin blocks were cut to a 60–80-nm thickness on an ultramicrotome (Leica UC7, Germany) and stained with 2% uranium acetate and 2.6% lead citrate. The longitudinal orientation of the inner retina was observed by TEM (HITACHI, HT7800, Japan), and images were captured.

### RGCs counting

To accurately count RGCs, we used RBPMS as a phenotypic marker, as reported in a previous study [40]. Freshly collected eyes were fixed in 4% paraformaldehyde for 2 h, and then the retina was isolated under an operating microscope. The retinal whole mounts were permeabilized with 0.5% Triton X-100 in PBS for 30 min and blocked in 10% normal goat serum for 1 h at room temperature, followed by incubation with anti-RBPMS antibody (1:200, Abcam, ab152101) overnight at 4 °C. The retinal sections were incubated with Alexa Fluor 594-conjugated secondary antibodies (1:100, Jackson Immunoresearch, 711-585-152) for 2 h at room temperature. Images were acquired using a 200x objective on a fluorescence microscope (Nikon, Tokyo, Japan).

### Electroretinography examination

RGCs function was assessed with the scotopic threshold response (STR) [56] and pattern electroretinogram (PERG) [57]. Following dark adaptation for over 16 h, the rats were anaesthetized, and the pupils were dilated with 0.5% atropine. Lidocaine hydrochloride eye drops were used before fixing the cornea-touch electrode. A subdermal ground electrode was fixed at the base of the tail, and the subdermal reference electrode was inserted at the forehead midway between the eyes. The STR responses of the rats were stimulated by flashes of light ranging from −6.5 to −4.5 log cd·s/m^2^, which were generated by a Ganzfeld stimulator (Roland Consult, Brandenburg, Germany) with an extra optical filter. The emergence of a positive and negative STR (pSTR/nSTR) was simultaneously recorded and analyzed with Roland RETI port/scan 21 equipment. The PERG responses were acquired by positioning rats 10 cm from the display monitors (Roland Consult, Brandenburg, Germany).

### Statistical analysis

All data are presented as the mean ± standard deviation (SD). The data were analyzed using SPSS 22.0 software. Differences among multiple groups were assessed by independent-sample two-tailed *t* tests. *p* < 0.05 was considered significant.

## Supplementary information

Supplementary Tables and Figures

ms data

## Data Availability

The data generated or analyzed during this study are included in this published article and its supplementary information files.
