# Supplementary material for: Sirt5-mediated desuccinylation of OPTN protects retinal ganglion cells from autophagic flux blockade in diabetic retinopathy
Source: Cell Death Discov. 2022 Feb 14;8:63. doi: 10.1038/s41420-022-00861-5 (PMC8844082; doi:10.1038/s41420-022-00861-5)
Supplement: Supplementary file 1 — Supplementary Tables and Figures [file 41420_2022_861_MOESM1_ESM.doc]

Supplementary Table 1. The modified site feature sequence and its enrichment statistics

| **Motif** | **Motif Score** | **Foreground** | | **Background** | | **Fold Increase** |
| --- | --- | --- | --- | --- | --- | --- |
|  |  | **Matches** | **Size** | **Matches** | **Size** |  |
| xxxxxxxxxx_K_Pxxxxxxxxx | 16.00 | 83 | 402 | 34267 | 603617 | 3.6 |
| xxxxxxxxxK_K_xxxxxxxxxx | 8.88 | 59 | 319 | 45432 | 569350 | 2.3 |
| xxxxxxxxxx_K_xxxxCxxxxx | 6.74 | 21 | 260 | 10901 | 523918 | 3.9 |

Supplementary Table 2. Polypeptide information sheet.

| Name | Sequences | Modified type | weight |
| --- | --- | --- | --- |
| Peptide 1 | CENERL-(succinyl)K-EELGK | succinyl-modified peptide | 1547.61 |
| Peptide 2 | SHENERL-(succinyl)K-EELGKC | succinyl-modified peptide | 1771.83 |
| Peptide 3 | CENERLKEELGK | nonmodified control peptide | 1447.61 |

Supplementary Table 3. The rabbit anti-OPTN-succinyl-K108 antibody ELISA results.

| Dilute concentration | Antibody purified from R2 (Ab2) | | | Antibody purified from R3 (Ab3) | | |
| --- | --- | --- | --- | --- | --- | --- |
|  | Peptide 1 | Peptide 2 | Peptide 3 | Peptide 1 | Peptide 2 | Peptide 3 |
| 1:6K | 2.037 | 2.105 | 0.084 | 2.044 | 1.916 | 0.102 |
| 1:18K | 2.015 | 2.062 | 0.097 | 2.035 | 1.926 | 0.093 |
| 1:54K | 1.647 | 1.916 | 0.071 | 1.86 | 2.097 | 0.067 |
| 1:162K | 1.126 | 1.289 | 0.087 | 1.382 | 1.649 | 0.082 |
| 1:486K | 0.535 | 0.624 | 0.061 | 0.728 | 1.108 | 0.061 |

**
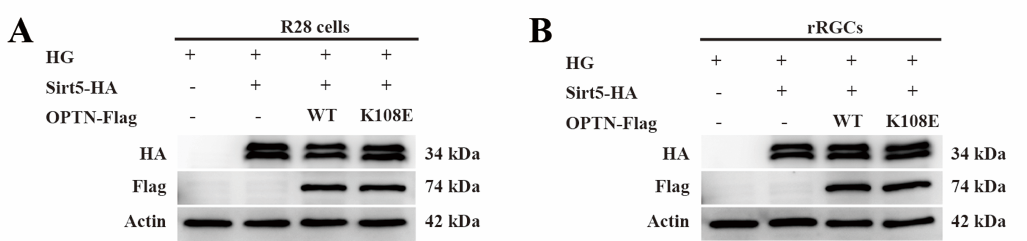
****Supplementary Figure 1. (A and B)** **The verification of transfection efficiency in R28 cells or rRGCs.** Cells were transfected with the indicated plasmids under high-glucose (HG) conditions (25 mM D-glucose), and transfected protein was detected and normalized with anti-Flag and anti-HA antibodies.

**
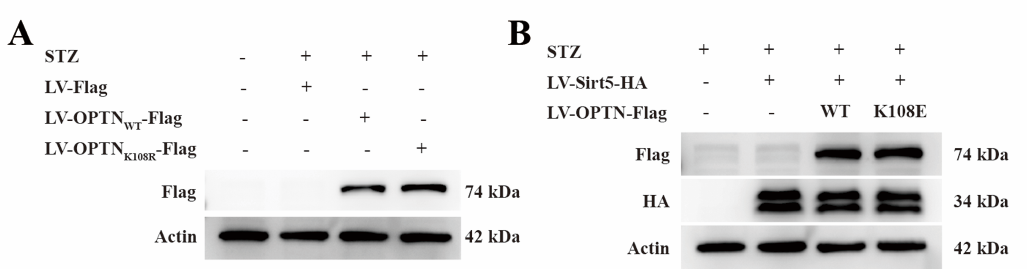
**

**Supplementary Figure 2**. **(A and B)** The verification of transfection efficiency. Rats with STZ-induced diabetes received intravitreal injections of indicated lentiviral (LV) particles.

**Supplementary Figure 3. The rabbit anti-OPTN-succinyl-K108 antibody dot blot results.** Different doses of modified peptides and non-modified peptides were fixed on the solid phase membrane. After incubation, enzyme-conjugated secondary antibody and chemiluminescence substrate were applied to detect the binding of polypeptide and antibody. The results showed that the Dot blot detection limit of Ab2 and Ab3 antibodies was up to 16ng, and non-modified polypeptides were almost not recognized.

a, OPTN K108su peptide 1; b, OPTN K108su peptide 2; c, non-modified control peptide 3
